# Medication errors in type 2 diabetes from patients’ perspective

**DOI:** 10.1371/journal.pone.0267570

**Published:** 2022-04-28

**Authors:** Julia K. Mader, Felix Aberer, Kerstin Sarah Drechsler, Tina Pöttler, Katharina M. Lichtenegger, Wolfgang Köle, Gerald Sendlhofer

**Affiliations:** 1 Department of Internal Medicine, Division of Endocrinology and Diabetology, Medical University of Graz, Graz, Styria, Austria; 2 Department of General Otorhinolaryngology, Medical University of Graz, Graz, Austria; 3 Medical Directorate, University Hospital of Graz, Graz, Styria, Austria; 4 Executive Department for Quality and Risk Management, University Hospital of Graz, Graz, Styria, Austria; 5 Department of Surgery, Research Unit for Safety and Sustainability in Healthcare, c/o Division of Plastic, Aesthetic and Reconstructive Surgery, Medical University of Graz, Graz, Styria, Austria; Imam Abdulrahman Bin Faisal University, SAUDI ARABIA

## Abstract

**Introduction:**

Drug errors pose a major health hazard to a number of patient populations. However, patients with type 2 diabetes mellitus seem especially vulnerable to this risk as diabetes mellitus is usually concomitant with various comorbidities and polypharmacy, which present significant risk factors for the occurrence of drug errors. Despite this fact, there is little data on drug errors from patients’ perspective. The present survey aimed to examine the viewpoints of patients with type 2 diabetes mellitus regarding their experiences with medication errors, the overall treatment satisfaction, and their perceptions on how a medication error was handled in daily hospital routine.

**Materials and methods:**

Inpatients at the Department of Endocrinology and Diabetology of the University Hospital of Graz were included in the survey. Out of 100 patients, one-half had insulin therapy before hospitalization while the other half had no insulin therapy prior to admission. After giving informed consent, patients filled out a questionnaire with 22 items.

**Results:**

Independent of their preexisting therapy, 25% of patients already suffered at least one drug error, whereby prescribing a wrong dose seemed to be the most common type of error. Furthermore, 26% of patients in the non-insulin versus 50% in the insulin group (*p = 0*.*084*) were convinced that drug errors were addressed honestly by the medical staff, while 54% in the non-insulin versus 80% in the insulin-group (*p = 0*.*061*) assumed that adequate measures were taken to prevent drug errors. Finally, 9 out of 10 patients seemed satisfied with their treatment regardless of their diabetes therapy.

**Discussion/conclusion:**

The results of the survey clearly showed that patients experienced at least one medication error during hospitalization. However, these errors only rarely led to patient harm. The survey also revealed the value of an honest and respectful doctor-patient relationship regarding patient perception of medication errors and general complaints. Increasing patient awareness on the existing in-hospital error management systems could eliminate treatment-related concerns and create a climate of trust that is essential for effective treatment.

## Introduction

Therapy-associated errors such as medication errors are becoming increasingly common in public perception. Today consensus exists that medication errors pose a serious risk to the well-being of patients, which is underestimated both in terms of incidence and in terms of the impact of errors. Therefore, increasing efforts are being made to investigate their causes and effects. According to a prospective cohort study by Barker, errors occur in up to 20% of all medication-related processes, with the most frequent being drug administration at the wrong time (43%), no administration of a prescribed medication (30%), and wrong dose administration (17%) [1]. Healthcare professionals may make these errors but also patients who are self-administrating medications. Thereof, 7% of all medication errors have the potential to cause adverse drug events [1].

Up to now, there has been no consistent definition of medication errors in the scientific literature and consequently the incidence of medication errors varies from five to 967 per one thousand patient days [2–4]. The World Health Organization, for example, defines medication error as “*a preventable event that may cause or lead to inappropriate medication use or patient harm while the medication is in the control of the healthcare professional*, *patient*, *or consumer*” [5–7]. Conversely/Similarly, the British psychologist James Reason postulated a system-oriented error theory with the help of the "Swiss cheese model of system accidents" [8]. According to his theory, risks and errors only occur when several safety barriers, which can be both human and technical, fail simultaneously. This means that the faulty system barriers are by no means rigid, but change over time [9].

A prominent risk factor for the occurrence of medication errors could be a lack of work experience [10]. Nurses with little work experience as well as doctors at the beginning of their career are at an increased risk of making a medication error. A study examining the theoretical and practical pharmacological knowledge of undergraduate students from seventeen European countries revealed that 46% of pharmaceutical therapies established by undergraduate students were wrong and that 55% of prescriptions contained at least one error. The most frequent error was choosing the wrong active agent or prescribing a wrong, usually too high, dosage. Even students in the final year of their training did not feel confident enough to prescribe medication safely and correctly [11]. Moreover, 30% of first-year physicians rated their knowledge of clinical pharmacology and pharmacotherapy as insufficient, while only 8% evaluated their knowledge as good [12–14].

Although diabetes mellitus is not an independent risk factor for the occurrence of medication errors, patients with diabetes mellitus are at higher risk to medication errors than others [15] due to the fact that antihyperglycaemic drugs, insulin in particular, belong to the group of so-called high-alert medications [16–20]. Furthermore, despite diabetes mellitus being a complex and widespread disease of great concern not only to those affected but also to their environment, including healthcare professionals, to date there are hardly any data on medication errors from the patients’ point of view. Therefore, the aim of the study was to survey hospitalized patients with type 2 diabetes mellitus (T2D) in terms of their perception of error management, therapy-associated satisfaction, and diabetes-specific treatment aspects during their hospital stay.

## Materials and methods

### Ethics statement

The study was approved by the Ethics Committee of the Medical University of Graz (submission number 29–278 ex 16/17). All participants gave their written consent to participate in the survey before any study-related procedures took place. The study was performed at the University Hospital of Graz in full accordance with the principles of the “Declaration of Helsinki” and Good Scientific Practice.

### The questionnaire

Two diabetologists, a diabetes educator, and a medical student developed the questionnaire in German language. The questionnaire was pretested in a small sample (n = 10) of individuals with type 2 diabetes from the outpatient clinic for relevance and comprehensibility and to remove any ambiguity which could affect question response. The feedback was integrated into the final version of the questionnaire. The questionnaire was completed in German only and was translated for publication (S1 and S2 Tables).

### Study design and setting

This single-center survey was carried out from April 2017 to February 2019 at the ward of the Division of Endocrinology and Diabetology. The subjects included a hundred patients with T2D: of these, fifty patients were on oral antihyperglycaemic agents (OAH) and the other fifty were on preexisting insulin therapy (with or without OAH). The classification of the two groups was based on individual treatment regimen prior to inpatient admission independent of the glucose-lowering therapy the patients received during their hospital stay. The patients were randomly recruited and those who met all inclusion (≥ 18 years of age, type 2 diabetes mellitus with pre-existing insulin therapy or pre-existing therapy with oral antidiabetic drugs) and none of the exclusion (type 1 diabetes mellitus, gestational diabetes, other forms of diabetes mellitus, patient unable to provide informed consent) criteria were included in the survey. Furthermore, demographic data (age, sex), height, weight and health data (diabetes duration, diabetes-specific therapy, HbA1c value from the routine laboratory), were also recorded. The patients were asked to complete a 22-item paper-based questionnaire (Table 1) which was collected after completion by study staff. Data, which were collected on the paper-based questionnaire, were manually entered into a digital database. All database entries were independently double-checked for correctness by two study team members. To obtain usable answers, only patients fluent in German were invited to participate in the survey.

**Table 1:** Baseline characteristics.

*Group*		*Female (%)*	*Age (years)*	*Diabetes duration (years)*	*HbA1c (mmol/mol)*	*BMI (kg/m* ^*2*^ *)*
**OAH**	Mean (SD)	38	67.9 ± 11.9	5.9±6.5	63 ± 25	29.8 ± 6.8
	Min.		42	0	38	21.5
	Max.		89	25	127	50.2
**Insulin (w./w.o. OAH)**	Mean (SD)	48	71.1 ± 11.9	20.0 ± 10.3	66 ± 20	31.0 ± 6.4
	Min.		34	1	40	16.7
	Max.		89	55	128	50.2

### Data collection

Items 1 to 8 of the questionnaire dealt with general aspects of medication errors such as fear of receiving a wrong medication as well as methods to avoid medication errors. Items 9 to 12 focused on the personal experience with medication errors. Items 13 to 17 dealt with therapy-associated patient satisfaction. Finally, items 18 to 22 of the questionnaire dealt with aspects associated with diabetes therapy.

For most items four answering categories existed: "yes", "rather yes", "rather no" and "no". Only items 6 and 8 involved open-ended questions. Details on questionnaire items are shown in the “S1” and the “S2” Tables.

### Statistical analysis

The COSMIN methodology was used for sample size estimation [21]. Furthermore, a quantitative survey was used to identify relevant content for a patient-reported outcome measure (PROM), while the survey sample size should be large enough to assume that saturation is reached. According to COSMIN recommendations, a very good rating is given if the sample size is at least one hundred. Therefore, an overall sample size of one hundred participants with fifty participants per subgroup (insulin-treated vs. insulin-naïve patients with T2D) was chosen.

Descriptive statistics (mean, standard deviation (SD)), frequency, and percentages were applied to all questionnaire items. Statistical data analysis was carried out by cross-tabulations (Chi2-test, Software: IBM SPSS Statistics, version 27, International Business Machines Corporation (IBM), USA). The significance level was set at α = 5%. All tests were two-sided and a p-value <0.05 was regarded as statistically significant.

## Results

Patients in the insulin group were older and had diabetes longer as compared to the OAH group. The proportion of women in the OAH group was 38% (n = 19) compared to 48% (n = 24) in the insulin group. The full set of baseline characteristics can be found in Table 1. Complete questionnaire results are shown in Tables 2–4.

**Table 2:** Beliefes on medication errors (group I = insulin; group O = oral antihyperglycaemic agent). Differences were calculated between both groups (O/I), duration of diabetes (D), age (A), gender (G) and education level (E). Any significance is given in the column “p-value” (n.s. = not significant). Significant differences for duration of diabetes, age, gender and education level are indicated in the description below the table.

Item	Yes (%)		Rather yes (%)		Rather no (%)		No (%)		No answer (%)		p-value
	O	I	O	I	O	I	O	I	O	I	
**1:** received wrong medication^#^	26	22	2	0	0	4	72	74	0	0	n.s.
**2:** received another patient’s medication ^#^	0	0	0	0	0	0	100	100	0	0	n.s.
**3:** hospital tries to prevent medication errors^#^	36	60	18	20	32	12	12	8	2	0	n.s.
**4:** medication errors are reported in a system^#^	18	36	20	18	30	18	22	24	10	4	n.s.
**5:** medication errors are openly addressed^#^	10	32	16	18	28	18	36	26	10	6	n.s.
**6:** what are reasons for medication errors	Open-ended question										
**7:** electronic prescribing can prevent medication errors^#^	16	22	12	20	20	16	40	36	12	6	n.s.
**8:** what measures should be taken to avoid errors	Open-ended question										
**9:** worried about receiving wrong medication^#^	14	10	14	12	10	14	62	64	0	0	n.s.
**10:** are concerns taken seriously^#^	44	52	32	30	20	14	4	2	0	2	n.s.
**11:** due to fear, did you stop medication intake^#^	34	38	4	2	6	6	56	54	0	0	n.s.
**12:** refused to take medication due to fear of an error^#^	14	18	8	4	12	10	66	68	0	0	n.s.

^#^For items 1–5, 7, 9–12, no statistically significant difference was observed for D, A, G, and E.

**Table 3:** Therapy associated satisfaction (group I = insulin; group O = oral antihyperglycaemic agent). Differences were calculated between both groups (O/I), duration of diabetes (D), age (A), gender (G) and education level (E). Any significance is given in the column “p-value” (n.s. = not significant). Significant differences for duration of diabetes, age, gender and education level are indicated in the description below the table.

Item	Yes (%)		Rather yes (%)		Rather no (%)		No (%)		No answer (%)		p-value
	O	I	O	I	O	I	O	I	O	I	
**13:** satisfied with physician’ care^#^	64	66	22	28	12	6	0	0	2	0	n.s.
**14:** satisfied with nursing care^#^	68	66	18	28	10	6	2	0	2	0	n.s.
**15:** concerns and questions were explained to you^#^	48	54	24	32	18	2	8	12	2	0	n.s.
**16:** received information about medication for intake^#^	36	54	34	22	22	16	8	8	0	0	n.s.
**17:** informed myself about medication intake^#^	54	60	8	6	10	10	28	24	0	0	n.s.

^#^For items 13–17, no statistically significant difference was observed for D, A, G, and E.

**Table 4:** Diabetes specific aspects (group I = insulin; group O = oral antihyperglycaemic agent). Differences were calculated between both groups (O/I), duration of diabetes (D), age (A), gender (G) and education level (E). Any significance is given in the column “p-value” (n.s. = not significant). Significant differences for duration of diabetes, age, gender and education level are indicated in the description below the table.

Item	Yes (%)		Rather yes (%)		Rather no (%)		No (%)		No answer (%)		p-value
	O	I	O	I	O	I	O	I	O	I	
**18:** blood glucose level is adjusted appropriately^#^	40	40	10	20	24	14	26	26	0	0	n.s.
**19:** afraid of hypoglycaemia^#^	8	32	6	10	8	8	76	50	2	0	O/I: 0.023
**20:** hypoglycaemia after self-administration	6	56	-	-	-	-	94	44	-	-	O/I: <0.001
**21:** hypoglycaemia after OAH^#^ treatment by physician/nurse	2	6	-	-	-	-	98	94	-	-	n.s.
**22:** hypoglycaemia after insulin treatment by physician/nurse	4	30	-	-	-	-	96	70	-	-	O/I: 0.001 G: 0.047

^#^For items 18–19, and 21 no statistically significant difference was observed for D, A, G, and E. For item 20, D: p<0.001, G: p = 0.041. For item 22, G: p = 0.016, G: p = 0.047.

### Patient perception of medication errors

For item 1, 28% (n = 14) of patients in the OAH group reported that they already experienced a medication error at least once during hospitalisation compared to 22% (n = 11) in the insulin group (p = 0.365). In addition, only 38% (n = 19) in the non-insulin group were aware of the existence of error reporting systems such as the critical incident reporting system compared to 54% (n = 27) of patients in the insulin group. Furthermore, patients who suffered from at least one medication error indicated that the error was caused by a wrong dose (OAH: n = 6 vs. insulin: n = 7) or a wrong drug/drug combination (OAH: n = 8 vs. insulin: n = 4). The reasons for medication errors (item 6) were answered manifold, with the following being assumed most often: work stress (OAH: 24% vs. insulin: 25%), staff shortage (OAH: 11% vs. insulin: 9%), distraction (OAH: 8% vs. insulin: 6%), human error (OAH: 4% vs. insulin: 7%) or communication failure (OAH: 6% vs. insulin: 1%).

The question on which measures should be taken to increase safety was answered by more respondents in the OAH group, (46%, n = 23) then in the insulin group (44%, n = 22). In this context, i) the desire for more information i.e., information about the therapy in general, the mode of drug action or potential adverse drug reactions, ii) more interaction with the physician or the nursing staff or iii) the wish for more time for patients’ needs was chosen most frequently. Furthermore, i) demand for more staff, ii) the desire for clear and more open communication both within the treating team and between patients and medical staff and iii) the desire for an open-minded patient safety culture were also mentioned.

### Therapy associated satisfaction

86% (n = 43) of patients in the OAH group and 94% (n = 47) in the insulin group were generally satisfied with physician care (item 13) during their hospital stay (p = 0.498). The same results (item 14) were achieved with regard to the patients’ opinions about the nursing staff (p = 0.463). Furthermore, 72% (n = 36) of patients in the OAH group and 86% (n = 43) in the insulin group were satisfied with the explanation of any drug-related question (p = 0.073). Finally, 62% (n = 31) of patients in the OAH group and 66% (n = 33) in the insulin group reported that they searched the web or asked their family doctor for more specific information regarding their medication (p = 0.929).

### Diabetes-specific therapy concerns

50% (n = 25) of patients in the OAH group and 60% (n = 30) in the insulin group were convinced that their blood glucose level (item 18) was in the target range (p = 0.394). In the insulin group, 42% (n = 21) were afraid of hypoglycaemia as compared to only 14% (n = 7) in the OAH group (p = 0.023). Only 6% (n = 3) of patients in the OAH group, assumed that hypoglycaemic events during hospitalization occurred due to diabetes self-management (insulin dose calculation and insulin administration) versus 56% in the insulin group (n = 28) who also shared that belief (p<0.001). Additionally, diabetes duration correlated with the occurrence of hypoglycaemia: 11% of patients with diabetes duration shorter than 10 years indicated that they already experienced hypoglycaemia caused by self-administration of antihyperglycaemic medication, compared to 53% with diabetes duration longer than 10 years (p<0.001). In the insulin group, women were 42% (n = 21) more likely to report having experienced hypoglycaemia at least once (p = 0.041).

## Discussion

This survey evaluated for the first time the perceptions of patients with T2D concerning medication errors, therapy associated satisfaction and diabetes-specific therapy concerns during hospitalization. So far, questionnaires on medication errors assessed only the attitude of healthcare providers towards error reporting while the patient perspective was not considered [22–24]. As negative outcome of medication errors directly affects patients it is of utmost importance to also assess patients’ perception on medication errors. To better address patients’ fears of medication errors in an inpatient setting where they are not in control of their medication, we need to understand their view on HCPs’ awareness, reporting and prevention of medical errors. Additionally, communication strategies to lay people on already established preventive error management can be adjusted to avoid knowledge gaps and fake news. Although patients with insulin-treated diabetes are classically regarded as high-risk with regard to the occurrence of medication errors, in this study no significant differences emerged with respect to medication errors when compared to patients on oral antihyperglycaemic agents alone [15–20]. In both groups, 25% of patients stated having experienced a medication error at least once. Most frequently, errors such as prescribing the wrong dosage or agent was mentioned. However, in line with the results of recent studies, only a small number of medication errors led to serious harm, while a large proportion of those had no lasting effect on patients’ wellbeing.

Of the cohort of patients with preexisting insulin therapy, only 26% assumed that medication errors that occurred were openly addressed. At the same time, slightly more than half of respondents in the OAH group (54%) believed that enough was done to avoid medication errors. Therefore, it can be assumed that there is still a chance to increase patients’ confidence in error prevention within the hospital. A survey of hospital pharmacists on medication error disclosure found, that the majority agreed that open communication is essential but differences in communication details and behavior when communicating to the patient (quote: “to give it a positive spin”) were described. Additionally, the response rate to this survey was low potentially having caused a positive selection bias overrepresenting pharmacists preferring open communication after occurrence of a medication error [25].

However, regardless of the preexisting, form of therapy the majority of surveyed patients, were not concerned about experiencing a medication error and felt that they were taken seriously enough when they expressed concerns about their medication. Furthermore, patients treated with OAH seemed to be more critical of the intra-hospital medication management than patients with insulin treated T2D, even if the differences were not statistically significant. Furthermore, patients undergoing insulin therapy were significantly more afraid of hypoglycaemic events than patients treated with OHAs. This is probably due to the fact that patients undergoing insulin therapy have already experienced hypoglycaemic episodes in the past. In addition, patients with longer diabetes duration feared hypoglycaemia more often than patients with a shorter duration of the disease. This finding may be explained by the fact that these patients were more often already receiving insulin therapy. Finally, women reported more frequently to having previously experienced hypoglycaemic episodes, which is in line with studies researching gender differences in hypoglycaemia in T2D [26]. This might also explain the greater fear of hypoglycaemia in females seen in our survey.

The fear of being subjected to a medication error has so far received little attention as a risk factor for non-adherence. In the present survey, nearly 25% of all patients—irrespective of the preexisting treatment type—indicated that they have refused to take medication at least once due to the fear of being subjected to a medication error. Chronic health conditions such as diabetes mellitus often require years of conscientious medicating to achieve the best possible therapeutic success. However, according to conservative estimates, more than 50% of all patients today do not take their medication as prescribed and thus jeopardize their own therapeutic success. The reasons for non-adherence found in the literature include inadequate health literacy, a challenging relationship between physicians and patients (especially in the sense of a paternalistic approach on the part of physicians), complex therapy regimes, communication problems, inadequate information on adverse drug events or limited access to healthcare [27]. A large majority of the surveyed patients saw stress as well as medical staff shortages as the root causes of medication errors. In addition, the lack of control mechanisms, multitasking, poor communication and similarities in drug name and design were mentioned as important medication errors triggers. Drugs with similar names [28, 29] or with similar appearance [30, 31] are a well-recognized risk factor for the occurrence of medication errors. It is estimated that between 7 and 20% of all medication errors occur due to similarly sounding name or similar physical shape of the drug [31].

In general, the vast majority of surveyed patients felt confident in their daily clinical routine and did not make any concrete suggestions for improvement. However, their desire to communicate more with the medical professionals as a measure to increase their subjective sense of safety in the treatment seemed to be a global concern. This finding is of great importance since the equally frequent request for more healthcare professionals is certainly less easy to accommodate than the desire for more time for medical consultation and education about disease and therapy. Overall, the survey revealed that, above all, improving the interpersonal relationship aspects (for example, appreciativeness, open-mindedness, etc.) helps to increase the patients’ personal sense of safety. Today, it is believed that good doctor-patient communication not only positively impacts the patient’s subjective feelings of wellbeing and their confidence, but may also improve compliance and in turn reduce the risk of medication errors [32]. Finally, the value of good communication is clearly underlined by the fact that most complaints about physicians are not based on their professional competence but on communication problems [33]. This is particularly important since it has been shown that patients who feel well looked after and adequately informed submit significantly fewer complaints and experience even less medication errors or complications, which is certainly a great advantage from a medical point of view [34].

## Conclusions

The survey results clearly show that patients experienced at least one medication error during hospitalization, however, those errors only rarely led to patient harm. The survey also highlighted the value of an honest and respectful doctor-patient relationship regarding the perception of medication errors or complaints in general. In addition, the fact that the patients’ confidence in in-hospital medication management can still be improved, it also seems to be important for day-to-day clinical routine, since many patients assume that errors are more likely to be swept under the rug than openly addressed. Increasing patient education on in-hospital error management systems could eliminate these concerns and create a climate of trust that is essential for effective treatment.

### Limitations

This study has several limitations. The survey was a monocentric study. Accordingly, the general significance of the survey may be limited. In addition, the survey was conducted at a tertiary care center, so the patient collective may be limited when compared to patient groups in primary or secondary centers. A further limitation may be that only patients fluent in German were included, which may have influenced or biased data. Finally, as with all surveys, there may have been biased answers due to a tendency of respondents to provide socially desirable answers. This particularly concerns the questions on own therapy adherence and therapy satisfaction.

## Supporting information

S1 Table Questionnaire (English translation of the questionnaire). (DOCX)

S2 Table Fragebogen (original version of the questionnaire in German language). (DOCX)
