# Supplementary material for: Medication errors in type 2 diabetes from patients’ perspective
Source: PLoS One. 2022 Apr 28;17(4):e0267570. doi: 10.1371/journal.pone.0267570 (PMC9049508; doi:10.1371/journal.pone.0267570)
Supplement: S1 Table — (DOCX) [file pone.0267570.s001.docx]

**S1 Table 1: Questionnaire**

# Medication errors in type 2 diabetes: perspective of patients

**A survey**

Informed consent signed: date __ / __ /_______

**Demographic data of participant:** *(filled in by a physician)*

**Gender:** O male O female

**Birth date:** _____ / _____ / ____________ / (dd/mm/yyyy)

**Highest completed education:** O Secondary school
O Apprenticeship O High school O Univ. Applied Sciences O University Others O ____________

**Diabetes since:** _____ Year(s)

**Last HbA1c:** _____ mmol/mol (from the last hospital stay)

**Hight:** ________ cm **Weight:** ________kg **BMI:** __________kg/m^2^

**Current Diabetes-therapy:** O diet O OAD/GLP-1

O Insulin (+OADs)

**Medication 1:** ______________________ **Dosage 1:** __________________

**Medication 2:** ______________________ **Dosage 2:** __________________

**Medication 3:** ______________________ **Dosage 3:** __________________

**Medication 4:** ______________________ **Dosage 4:** __________________

**Insulin 1:** ______________________ **Dosierung 1:** __________________

**Insulin 2:** ______________________ **Dosierung 2:** __________________

**Insulin 3:** ______________________ **Dosierung 3:** __________________

**Investigator:** _____________________________________________________

1 = yes / 2 = rather yes / 3 = rather no / 4= no

| **N** | **Item** |
| --- | --- |
|  | **Perception on medication errors** |
| 1 | Have you ever received a wrong medication? |
| 2 | Have you ever received another patient’s medication by mistake? |
| 3 | Do you believe that hospitals try to prevent medication errors? Frage 17 |
| 4 | Do you believe that medication errors are objectively reported in the recording system? |
| 5 | Do you believe that medication errors are openly addressed? |
| 6 | What are, in your opinion, reasons for medication errors? |
| 7 | Do you believe that an electronic prescription system can help prevent the occurrence of medication errors? |
| 8 | What measures should be taken to avoid medication errors? |
| 9 | Are you worried about receiving a wrong medication during the hospital stay (for example, wrong drug, wrong dose)? |
| 10 | Are concerns (for example, therapy, medication, etc.) taken seriously, if they occur? |
| 11 | In terms of fear of side effects, have you ever stopped taking medication? |
| 12 | Have you ever refused to take your medication for fear of getting the wrong medication? |
|  | **Therapy-associated satisfaction** |
| 13 | Are you satisfied with the physicians’ care during your stay? |
| 14 | Are you satisfied with the nursing care during your stay? |
| 15 | Were your questions/concerns about your necessary medication sufficiently and understandably explained to you? |
| 16 | Were you adequately informed about the medications you are receiving? |
| 17 | Did you inform yourself about medications (for example family doctor, friends, internet, magazines etc.)? |
|  | **Diabetes-specific aspects** |
| 18 | Do you think that your blood glucose level is adjusted appropriately? |
| 19 | Are you afraid of hypoglycaemia? |
| 20 | Have you ever had hypoglycaemia by self-administering antihyperglycaemic agents? |
| 21 | Have you ever had hypoglycaemia after physician’s/nursing administration of oral antihyperglycaemic agents? |
| 22 | Have you ever had hypoglycaemia after physician’s/nursing administration of insulin? |
