# Supplementary material for: Medication errors in type 2 diabetes from patients’ perspective
Source: PLoS One. 2022 Apr 28;17(4):e0267570. doi: 10.1371/journal.pone.0267570 (PMC9049508; doi:10.1371/journal.pone.0267570)
Supplement: S2 Table — (DOCX) [file pone.0267570.s002.docx]

**S2: Fragebogen**

**Medikation aus Sicht von PatientInnen auf einer internistischen Normalstation – eine Fragebogenerhebung**

Einverständniserklärung unterschrieben am __ / __ /_______

**Demographische Daten des/ der Befragten:** *(durch Arzt auszufüllen)*

**Geschlecht:** O männlich O weiblich

**Geburtsdatum:** _____ / _____ / ____________ / (tt/mm/jjjj)

**Höchste abgeschlossene Ausbildung:** O Hauptschule O Lehre

O Matura O Fachhochschule O Universität O ____________

**Diabetes seit:** _____ Jahren

**Aktueller HbA1c:** _____ mmol/mol (aus dem stat. Aufenthalt)

**Größe:** ________ cm **Gewicht:** ________kg **BMI:** __________kg/m^2^

**Derzeitige Diabetestherapie:** O diätetisch O OAD/GLP-1

O Insulin (+OADs)

**Präparat 1:** ______________________ **Dosierung 1:** __________________

**Präparat 2:** ______________________ **Dosierung 2:** __________________

**Präparat 3:** ______________________ **Dosierung 3:** __________________

**Präparat 4:** ______________________ **Dosierung 4:** __________________

**Insulin 1:** ______________________ **Dosierung 1:** __________________

**Insulin 2:** ______________________ **Dosierung 2:** __________________

**Insulin 3:** ______________________ **Dosierung 3:** __________________

**Investigator:** _____________________________________________________

1 = Ja / 2 = eher Ja / 3 = eher Nein / 4= Nein

1) Sind Sie mit der ärztlichen Betreuung während Ihres Aufenthalts zufrieden?

2) Sind Sie mit der pflegerischen Betreuung während Ihres Aufenthalts zufrieden?

1. Werden Ihre Fragen zu Ihrer notwendigen Medikation ausreichend und verständlich erklärt?

4) Sind Sie über die Medikamente, die Sie erhalten, ausreichend informiert?

5) Informieren Sie sich selbst über Medikamente? (zum Beispiel Hausarzt, Freunde, Bekannte, Internet, Zeitschriften etc.)

6) Haben Sie Bedenken, im Krankenhaus falsche Medikamente verabreicht zu bekommen? (zum Beispiel falscher Wirkstoff, falsche Dosierung)

7) Wird auf Ihre Bedenken genügend eingegangen, sofern solche auftreten? (z.B. Therapie, Medikamente, etc.)

8) Haben Sie - aus Angst vor Nebenwirkungen - schon einmal auf die Einnahme von

Medikamenten verzichtet?

9) Haben Sie schon einmal abgelehnt, Ihre Medikamente einzunehmen aus Angst falsche Medikamente zu bekommen?

10) Haben Sie schon einmal falsche Medikamente erhalten?

11) Haben Sie schon einmal irrtümlich die Medikamente von Ihrem Zimmernachbarn/Ihrer Zimmernachbarin erhalten?

12) Glauben Sie, dass Ihr Blutzucker passend eingestellt ist?

13) Haben Sie Angst vor Hypoglykämien (=Unterzuckerungen)?

14) Hatten Sie schon einmal Hypoglykämien durch Selbstverabreichung von Medikamenten?

15) Hatten Sie schon einmal Hypoglykämien nach ärztlicher/pflegerischer Verabreichung von oralen Antidiabetika?

16) Hatten Sie schon einmal Hypoglykämien nach ärztlicher/pflegerischer Verabreichung von Insulin?

17) Glauben Sie, dass genügend unternommen wird, um Medikationsfehler zu vermeiden?

18) Glauben Sie, dass es ein System gibt, in dem Medikationsfehler objektiv gemeldet werden?

19) Glauben Sie, dass Medikationsfehler offen adressiert werden?

20) Was sind Ihrer Meinung nach die Ursachen für Medikationsfehler?

21) Glauben Sie, dass elektronische Fieberkurven das Auftreten von Medikationsfehlern vermeiden können?

22) Welche Maßnahmen sollen Ihrer Meinung nach getroffen werden, damit Sie sich sicherer fühlen?
